# *Aristolochia quangbinhensis* (Aristolochiaceae), a new species from Central Vietnam

**DOI:** 10.3897/phytokeys.33.6094

**Published:** 2014-01-22

**Authors:** Truong Van Do, Trong Duc Nghiem, Stefan Wanke, Christoph Neinhuis

**Affiliations:** 1 Institut für Botanik, Technische Universität Dresden, Zellescher Weg 20b, D–01062 Dresden, Germany; 2 Vietnam National Museum of Nature, Vietnam Academy of Science & Technology, 18 Hoang Quoc Viet, Hanoi, Vietnam; 3 Department of Botany, Hanoi University of Pharmacy, 13–15 Le Thanh Tong, Hanoi, Vietnam

**Keywords:** *Aristolochia*, *Aristolochia quangbinhensis*, Aristolochiaceae, *Isotrema*, new species, Vietnam

## Abstract

*Aristolochia quangbinhensis* T.V. Do, a new species from Central Vietnam, is described and illustrated. According to morphology, the species belongs to *Aristolochia* subgenus *Isotrema*. A detailed description, along with line drawings, photographs, ecology, distribution, conservation status as well as a comparison to morphologically similar species is provided.

## Introduction

*Aristolochia* comprises about 500 species and is the largest genus of Aristolochiaceae (Wagner et al. 2012). Recent phylogenetic studies of the genus based on morphological and molecular data suggested a subdivision of *Aristolochia* into three subgenera: *Aristolochia*, *Isotrema* and *Pararistolochia* (Wanke et al. 2006). *Aristolochia* subgenus *Aristolochia* occurs from the Mediterranean zone to subtropical and tropical areas of America, Africa and Asia, *Aristolochia* subgenus *Pararistolochia* is present in tropical Africa and Australasia, whereas *Aristolochia* subgenus *Isotrema* shows a disjunct Asian and Central- and North American distribution (Wanke et al. 2006, Ohi-Toma et al. 2006, González et al. 2013, Buchwalder et al. in press). *Aristolochia* subgenus *Isotrema* (hereafter shortened to *Isotrema*) comprises about 70 species, 50 of which occur in Asia (González et al. 2013). In Vietnam two subgenera occur, namely *Aristolochia* and *Isotrema*.

*Isotrema* is well known for its U- or horseshoe-shaped perianth, the utricle and the tube are not sharply delimited, a strongly folded or curved tube, a 3-lobed limb, sometimes with fused lobes, and a gynostemium consisting of three segments, each of them carrying two anthers on the outer surface. In contrast, subgenus *Aristolochia* can be recognized by its slightly curved or rectilinear tube, the utricle and the tube are sharply distinct, a one- to three-lobed perianth limb, a gynostemium with more than three lobes, each of them carrying a single anther on the outer surface. Based on these characters, the new species can be easily assigned to subgenus *Isotrema*.

In an illustrated Flora of Vietnam, Ho (2000) reported 11 species of *Aristolochia*, including four belonging to *Isotrema*. Hwang et al. (2003) listed 45 species of *Aristolochia* for the Flora of China, 33 of which are restricted to this country. In the latter study, 29 Chinese species belong to *Isotrema*, and only one of them (*Aristolochia petelotii* O.C. Schmidt) was mentioned to occur also in Vietnam, although the floras of southern China and northern Vietnam have many angiosperm species in common. Ban (2003) recorded 13 species and one variety for Vietnam, and listed the same species of *Isotrema* as those mentioned by Ho (2000), plus *Aristolochia kwangsiensis* W.Y. Chun & F.C. How ex C.F. Liang. In preparation of a taxonomic revision of *Aristolochia* for Vietnam, a new *Aristolochia* species belonging to *Isotrema* was found in Central Vietnam and is described here.

## Methods

Based on morphological characters, a first overview of the genus *Aristolochia* from Vietnam and adjacent areas (southern China, Laos, Cambodia & Thailand) was prepared. All available specimens of *Aristolochia* housed in Vietnamese herbaria (CPNP, HN, HNU, IMM, VNM VNMN), relevant collections from institutions abroad (DR, HITBC, IBK, IBSC, K, KUN, L, MO, P, SING) and material from recent fieldwork were examined. All morphological characters were studied under dissecting microscopes, and are described using the terminology presented by Harris (2001) & Hwang et al. (2003).

## Taxonomy

### Aristolochia quangbinhensis

T. V. Do sp. nov.

urn:lsid:ipni.org:names:77135668-1

http://species-id.net/wiki/Aristolochia_quangbinhensis

Figure 1 , 2

#### Note.

This new species is morphologically similar to *Aristolochia championii* Merill & W.Y. Chun, *Aristolochia vallisicola* T.L. Yao, *Aristolochia petelotii* O.C. Schmidt and *Aristolochia versicolor* S.M. Hwang, but is distinguishable from these species by the following diagnostic characters: petiole 1.5–2.5(–3) cm long; lamina elliptic to oblong-elliptic; peduncle 1.5–2 cm long, covered with yellow-brown trichomes; perianth limb bell-shaped, 2–2.5(–3) cm wide, exclusively purplish-pink on both sides, no blotches or veins are visible; perianth margins recurved; flower tube mouth slightly darker than the remaining perianth limb; perianth tube pale yellow to whitish and the entire back of the perianth limb and tube covered with yellow-brown trichomes.

#### Type.

VIETNAM. Quang Binh province: Minh Hoa district, Hoa Luong community, 17°47'5.00"N, 105°52'20.05"E, elev. 380 m, 3 April 2013, *T.V. Do* 39 (holotype: VNMN; isotype: DR).

#### Description.

Perennial woody lianas. Roots numerous, fasciculate and cylindrical. Stems terete, densely yellow-brown villous when young, older stems with corky bark, glabrous. Petiole 1.5–2.5(–3) cm long, straight, densely covered with yellow-brown trichomes; lamina elliptic to oblong-elliptic, (6–)8–13(–14) cm long, 3–5(–6) cm wide, subcoriaceous, base subcordate to auriculate, with a shallow sinus 3–4 mm deep, (1–)1.5–2 mm wide, apex acute, adaxially glabrous, dark green, abaxially densely yellow-brown villous, basal veins 3, palmate, secondary veins 7–8 pairs, pinnate, venation densely reticulate and prominent on both sides. Flowers terminal, solitary, but accompanied by a lanceolate scale-like bracteole, (2–)3 mm long, (1.5–)2 mm wide, sessile, inserted near base of the peduncle, conspicuous, persistent. Peduncles 1.5–2 cm long, pendulous, purple, densely hirsute. Peduncles, bracts and perianth densely covered by yellow-brown pluricellular hairs. Perianth S-shaped, (3–)3.2–3.5 cm long, outside densely yellow-brown hirsute with obscure, parallel veins, inside smooth. Ovary oblong, (0.8–)1–1.2 cm long, 0.3–0.4 cm diam., yellowish-green, densely hirsute, 6-locular, ovules numerous, usually in one series. Utricle ovoid, (1.2–)1.5–1.8 cm long, 0.5–0.6(–0.8) cm diam., externally white, inner surface basally with a dark-purple patch and distally with a white patch; tube strongly curved at its base, parallel and in close contact with the utricle, narrower than the utricle, cylindrical, 0.9–1.0 cm long, 0.4–0.5 cm diam.; limb three-lobed, bell-shaped, 2–2.5(–3) cm diam., purplish-pink on both sides, without any blotches or veins visible, outer surface densely hirsute, inner surface smooth, the three unequal lobes valvate in preanthetic flowers, the lateral lobes broadly deltoid with acute apex, the lower (median) lobe semicircular, during anthesis bell-shaped and with the margins recurved; throat circular, dark-purple, densely papillose; annulus present, formed by an ellipsoid flange, dark-violet; gynostemium three-lobed, lobes with obtuse apices, smooth, (3–)4–5 mm high, 2–3 mm diam., white; stamens six in one serie of three pairs; anthers oblong, (2–)2.5–3 mm long, yellow, tetralocular, longitudinally dehiscent. Capsules not seen.

**Figure 1.**
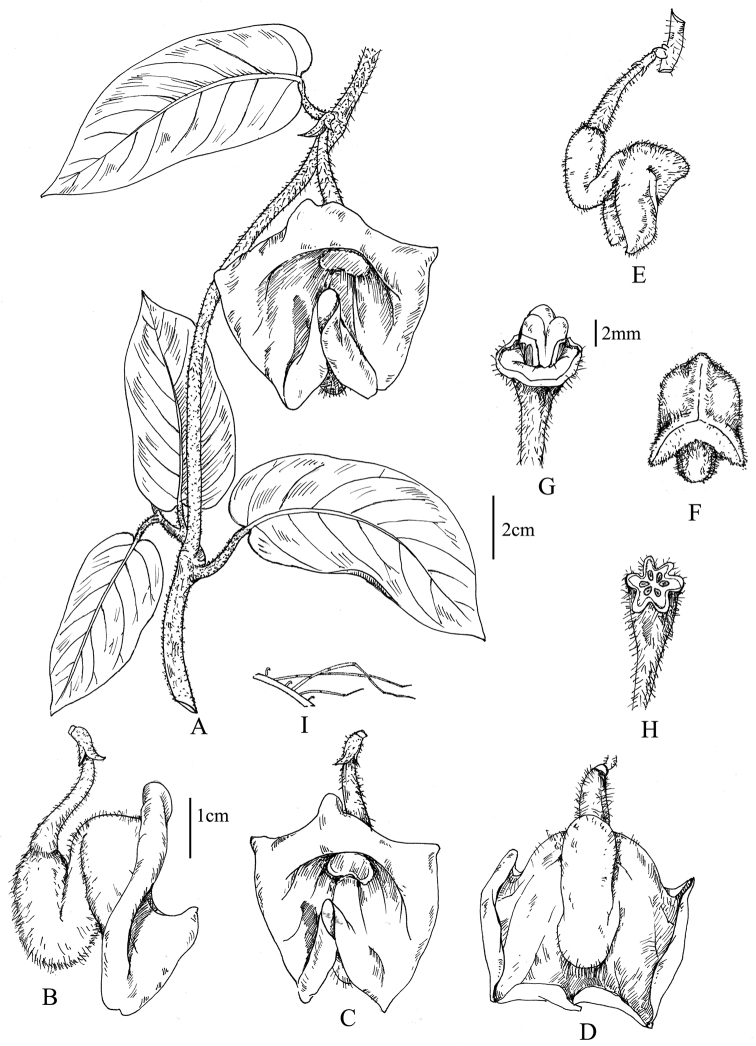
Line drawing of *Aristolochia quangbinhensis* T.V. Do. **A** Flowering branch **B–D** Lateral (**B**) frontal (**C**) and dorsal (**D**) views of a flower at anthesis **E–F** Lateral (**E**) and frontal (**F**) views of a preanthetic flower **G** Gynostemium and ovary **H** Transverse section of ovary **I** Multicellular trichomes on the surface of the petiole. Drawing by N.V. Quyet from the holotype.

**Figure 2.**
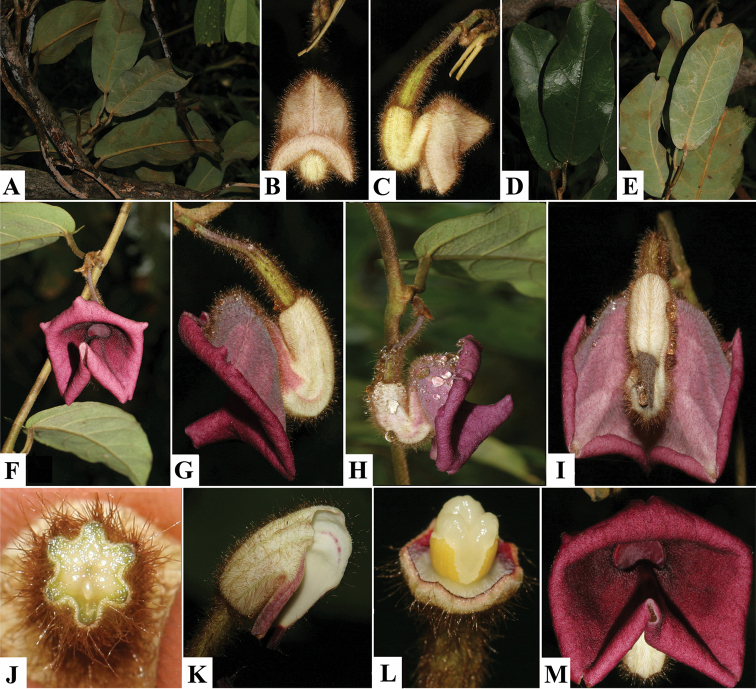
Photographs of *Aristolochia quangbinhensis* T.V. Do. **A** Habit **B–C** Frontal (**B**) and lateral (**C**) views of a preanthetic flower **D–E** Leaf in adaxial (**D**) and abaxial (**E**) views **F** Flowering branch **G–I** Lateral (**G–H**) and dorsal (**I**) views of flowers at anthesis **J** Transverse section of the ovary **K** Inner surface of perianth **L** Gynostemium and ovary **M** Frontal view of an anthetic flower. Photographs taken on the holotype locality in Hoa Luong community, Minh Hoa district, Quang Binh province, Central Vietnam.

#### Vernacular name.

Phòng Kỷ Quảng Bình (in Vietnamese).

#### Distribution.

**Figure 3.**
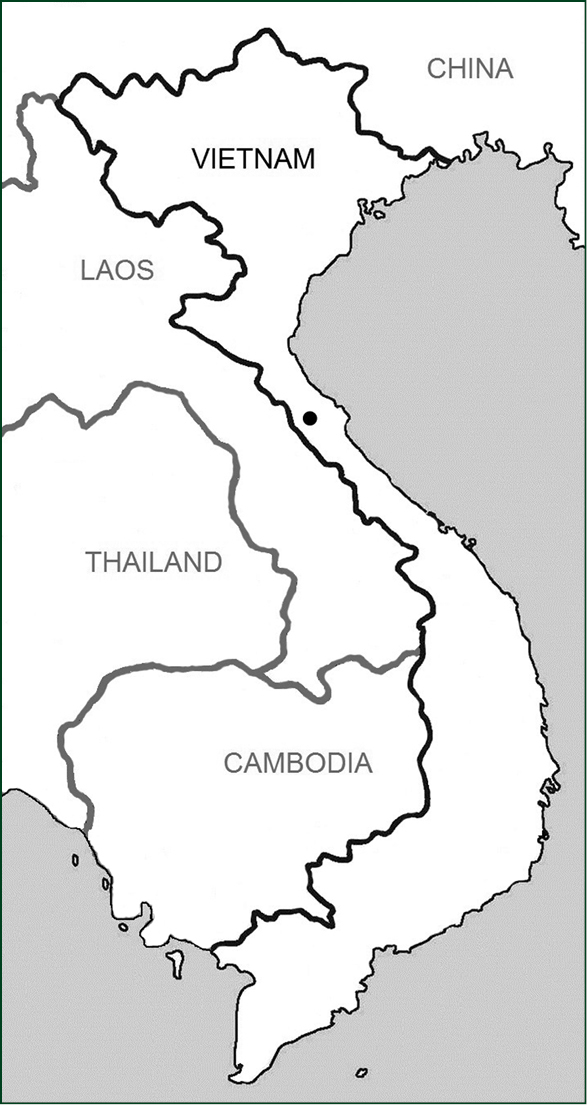
Distribution (dot) of *Aristolochia quangbinhensis* T.V. Do in Central Vietnam.

*Aristolochia quangbinhensis* is known from a single population found on the north-eastern slope of a mountain range bordering the buffer zone of Phong Nha-Ke Bang National Park in the Hoa Luong community, Minh Hoa district, Quang Binh province (Fig. 3). It might also be present in Laos P.D.R., because of the proximity of the Khammouan province with Central Laos.

#### Ecology.

*Aristolochia quangbinhensis* occurs in humid mountain sites, in the understory of disturbed lowland evergreen broad-leaved forest and mainly evergreen scrubs. Dominant plants at the type locality are Annonaceae (*Desmos* spp.), Apocynaceae (*Marsdenia* spp., *Wrightia* spp.), Fabaceae (*Millettia* spp.), Lauraceae (*Machilus* spp., *Litsea* spp.), Malvaceae (*Sterculia* spp.), Pandaceae (*Microdesmis* spp.), Phyllanthaceae (*Antidesma* spp., *Aporosa* spp., *Glochidion* spp.), and Rubiaceae (*Randia* spp.).

#### Phenology.

Flowering specimens have been collected in April and May but it is possible that blooming already begins in March.

#### Etymology.

The specific epithet refers to the type locality.

#### Conservation status.

In the past, large areas of primary, broad-leaved, evergreen forest covered the Hoa Luong community. Excessive logging, however, resulted in the loss of large extensions of primary forest. Although logging was prohibited in the 1990s, local farmers continued to impose strong pressure on the remaining forest patches converting it mostly into corn and soybean fields. As a result, the flora of the area should be regarded as threatened by extinction. Within the area, *Aristolochia quangbinhensis* is known from a single population; in fact, during the present study, only two healthy individuals were located growing about 50 m apart from each other. Therefore, the new species is assigned a preliminary status of vulnerable (VU D2) according to IUCN Red List criteria (IUCN 2013), indicating a population with a very restricted area of occupancy (typically less than 20 km^2^) or the number of locations (typically five or fewer) being both at hand for *Aristolochia quangbinhensis*. The lack of data currently does not allow a final risk evaluation, but the species might also be regarded as endangered (EN).

## Discussion

*Aristolochia quangbinhensis* is morphologically similar to *Aristolochia vallisicola* T.L. Yao (reported from Pahang, Peninsular Malaysia), *Aristolochia championii* Merr. et Chun (known from Guangdong, Guangxi, China), *Aristolochia versicolor* S.M. Hwang (reported from China and Thailand) and *Aristolochia petelotii* O.C. Schmidt (reported from Vietnam and China). However, the new species differs from the aforementioned species by several important vegetative and reproductive characters (summarized in Table 1). This new discovery, along with several new species recently described from Thailand (González and Poncy 1999, Phuphathanaphong 2006), Hainan Island, China (Han Xu et al. 2011), and Peninsular Malaysia (Yao 2012), provide evidence that the genus *Aristolochia* and in particular *Aristolochia* subgenus *Isotrema* is very diverse in South-East Asia. A detailed investigation of the different flower phenotypes and inflorescence between Asian, North and Central American *Isotrema* species is needed to reconstruct the evolution of floral forms between the biogeographic areas.

**Table 1.** Comparison between *Aristolochia quangbinhensis* and its four morphologically closest relatives.

**Characters**	***Aristolochia quangbinhensis***	***Aristolochia vallisicola***	***Aristolochia championii***	***Aristolochia versicolor***	***Aristolochia petelotii***
Petiole	1.5–2.5(–3) cm long, densely yellow-brown villous	2.5–7 cm long, puberulent	1–2.0 cm long, densely villous	1–2.0 cm long, sparsely pilose	2–4 cm long, densely yellow-brown villous
Lamina	elliptic to oblong-elliptic, (6 –)8–13(–14) cm long, 3–5(–6) cm wide	lanceolate, oblanceolate to broadly oblanceolate, 6.5–11 cm long, 1.7–3.9 cm wide	elliptic- lanceolate to linear-lanceolate, 15–30 cm long, 2–5 cm wide	narrowly elliptic to lanceolate-elliptic, 7.5–33 cm long, 4–12 cm wide	narrowly ovate to lanceolate ovateblade lamina, 12–20(–22) cm long, 5–11(–13) cm wide
Leaf base	narrowly auriculate; 3–4 mm deep	cordate; 2–3 mm deep	rounded to shallowly cordate; 2 mm deep	narrowly auriculate; 5–7 mm deep	shallowly cordate, sinus 6–10 mm deep
Leaf apex	acute	acute	acuminate	acute to acuminate	acuminate
Adaxial surface of the leaf	glabrous	glabrescent	glabrous but villous along veins	glabrous	glabrous
Abaxial surface of the leaf	densely yellow-brown villous	puberulent	densely brown villous	sparsely villous along veins, glaucous	pubescent
Inflorescences	ramiflorous, flower solitary	cauliflorous, flower solitary	cauliflorous, cluster of 2–5 flowers	ramiflorous, solitary or flower pair	cauliflorous, cluster of 2–3 flowers
Peduncle	1.5–2 cm long; densely hirsute, unbranched	15.5–17 cm long; puberulent, branched	3–4 cm long; brown villous, unbranched	2–3 cm long; brown villous, unbranched	10–12 cm long, densely brown villous, unbranched
Perianth	yellowish-white; (3–)3.2–3.5 cm long; without blotches	purple; 6–6.5 cm long; with obscure veins	greenish-yellow; 10–12 cm long; with purple veins and blotches	yellow-green; 7–9 cm long; with purple veins	yellow-purple, 8–10 cm long; with purple veins and blotches
Limb	purplish-pink; bell-shaped; 2–3 cm wide; unequal 3-lobed, margin of lobes recurved, acute apex	yellow; disc-shaped; 5.8–6.5 cm wide; equal 3-lobed, margin of lobes expanded, rounded apex	yellow; funnel-shaped; 4–6 cm wide; unequal 3-lobed, lower one spreading spathulate-like, margin of lobes erect, rounded apex	purple; disc-shaped; 4–6 cm wide; equal 3-lobed, margin of lobes expansive, rounded apex	yellow; bell-shaped; 4–5 cm wide; unequal 3-lobed, margin of lobes rolled downwards, acute apex
Throat	annulus present, throat dark-violet	annulus present, throat coloration unknown	annulus present, throat yellow	annulus absent, throat coloration unknown	annulus present, throat dark-purple
Distribution	Central Vietnam	Peninsular Malaysia	Southern China	Southern China, North Eastern Thailand	Southern China, Northern Vietnam

## Supplementary Material

XML Treatment for Aristolochia quangbinhensis
